# A modified clinicopathological tumor staging system for survival prediction of patients with penile cancer

**DOI:** 10.1186/s40880-018-0340-x

**Published:** 2018-11-23

**Authors:** Zai-Shang Li, Antonio Augusto Ornellas, Christian Schwentner, Xiang Li, Alcides Chaux, Georges Netto, Arthur L. Burnett, Yong Tang, JiunHung Geng, Kai Yao, Xiao-Feng Chen, Bin Wang, Hong Liao, Nan Liu, Peng Chen, Yong-Hong Lei, Qi-Wu Mi, Hui-Lan Rao, Ying-Ming Xiao, Qi-Lin Wang, Zi-Ke Qin, Zhuo-Wei Liu, Yong-Hong Li, Zi-Jun Zou, Jun-Hang Luo, Hui Li, Hui Han, Fang-Jian Zhou

**Affiliations:** 1 Department of Urology, Shenzhen People’s Hospital, The Second Clinical College of Jinan University, Shenzhen, 518060 Guangdong P. R. China; 2 Sun Yat-sen University Cancer Center; State Key Laboratory of Oncology in South China; Collaborative Innovation Center of Cancer Medicine, Guangzhou, 510060 Guangdong P. R. China; 3 Department of Urology, Sun Yat-sen University Cancer Center, 651 Dongfeng Road, East Guangzhou, 510060 Guangdong P. R. China; 4 Department of Urology, Brazilian National Institute of Cancer and Hospital Mário Kröeff, Rio de Janeiro, 20230-130 Brazil; 5 Section of Urology, National Institute of Cancer, Rio de Janeiro, Brazil; 6 Department of Urology, Diakonie Klinikum Stuttgart, 70174 Stuttgart, Germany; 7 Urological Department, Urological Institute, West China Hospital of Sichuan University, Chengdu, 610041 Sichuan P. R. China; 8 Department of Scientific Research, Norte University, 1001-1925 Asunción, Paraguay; 9 Department of Pathology, Johns Hopkins University, Baltimore, MD 21218 USA; 10 Department of Urology, Johns Hopkins University, Baltimore, MD 21218 USA; 11 Department of Urology, Affiliated Cancer Hospital of Guangxi Medical University, Nanning, 530021 Guangxi P. R. China; 12 Department of Urology, Kaohsiung Medical University Hospital, Kaohsiung, Taiwan, 807 China; 13 Department of Urology, The First People’s Hospital of Chenzhou, Chenzhou, 423000 Hunan P. R. China; 14 Department of Urology, Cancer Center of Guangzhou Medical University, Guangzhou, 510000 Guangdong P. R. China; 15 Department of Urology, Sichuan Cancer Hospital, Chengdu, 610041 Sichuan P. R. China; 16 Department of Urology Oncological Surgery, Chongqing Cancer Hospital & Institute & Cancer Center, Chongqing, 400040 China; 17 Department of Urology, Affiliated Tumor Hospital of Xinjiang Medical University, Urumqi, 830011 Xinjiang P. R. China; 18 Department of Urology, Yunnan Provincial Tumor Hospital, The Third Affiliated Hospital of Kunming Medical University, Kunming, 650118 Yunnan P. R. China; 19 Department of Urology, Dongguan People’s Hospital, Dongguan, 523059 Guangdong P. R. China; 20 Department of Pathology, Sun Yat-sen University Cancer Center, Guangzhou, 510060 Guangdong P. R. China; 21 Department of Urology, First Affiliated Hospital, Sun Yat-sen University, Guangzhou, 510000 Guangdong P. R. China; 22 Department of Pathology, The Chinese University of Hong Kong, Hong Kong, 999077 P. R. China

**Keywords:** Penile neoplasms, Lymph node metastasis, Staging, Lymph node excision, Prognosis

## Abstract

**Background:**

The 8th American Joint Committee on Cancer tumor–node–metastasis (AJCC-TNM) staging system is based on a few retrospective single-center studies. We aimed to test the prognostic validity of the staging system and to determine whether a modified clinicopathological tumor staging system that includes lymphovascular embolization could increase the accuracy of prognostic prediction for patients with stage T2–3 penile cancer.

**Methods:**

A training cohort of 411 patients who were treated at 2 centers in China and Brazil between 2000 and 2015 were staged according to the 8th AJCC-TNM staging system. The internal validation was analyzed by bootstrap-corrected C-indexes (resampled 1000 times). Data from 436 patients who were treated at 15 centers over four continents were used for external validation.

**Results:**

A survivorship overlap was observed between T2 and T3 patients (*P* = 0.587) classified according to the 8th AJCC-TNM staging system. Lymphovascular embolization was a significant prognostic factor for metastasis and survival (all *P* < 0.001). Based on the multivariate analysis, only lymphovascular embolization showed a significant influence on cancer-specific survival (CSS) (hazard ratio = 1.587, 95% confidence interval = 1.253–2.011; *P* = 0.001). T2 and T3 patients with lymphovascular embolization showed significantly shorter CSS than did those without lymphovascular embolization (*P* < 0.001). Therefore, a modified clinicopathological staging system was proposed, with the T2 and T3 categories of the 8th AJCC-TNM staging system being subdivided into two new categories as follows: t2 tumors invade the corpus spongiosum and/or corpora cavernosa and/or urethra without lymphovascular invasion, and t3 tumors invade the corpus spongiosum and/or corpora cavernosa and/or urethra with lymphovascular invasion. The modified staging system involving lymphovascular embolization showed improved prognostic stratification with significant differences in CSS among all categories (all *P* < 0.005) and exhibited higher accuracy in predicting patient prognoses than did the 8th AJCC-TNM staging system (C-index, 0.739 vs. 0.696). These results were confirmed in the external validation cohort.

**Conclusions:**

T2–3 penile cancers are heterogeneous, and a modified clinicopathological staging system that incorporates lymphovascular embolization may better predict the prognosis of patients with penile cancer than does the 8th AJCC-TNM staging system.

*Trial registration* This study was retrospectively registered on Chinese Clinical Trail Registry: ChiCTR16008041 (2016-03-02). http://www.chictr.org.cn

## Background

The American Joint Committee on Cancer tumor–node–metastasis (AJCC-TNM) staging system for penile cancer is widely used to predict patient prognoses, guide treatment, and evaluate treatment results at different centers [1, 2]. Compared with previous editions, the 7th AJCC-TNM staging system better predicts the prognosis of patients with penile squamous cell carcinoma [3–5]. However, previous studies on penile cancer indicated that patients with stage T2 and T3 cancer had similar survival outcomes [6], suggesting that the 7th AJCC-TNM staging system did not sufficiently differentiate patients with different prognoses [4, 7]. In 2017, T2 and T3 diseases were re-defined in the 8th AJCC-TNM staging system [1, 2]. However, the weakness of the AJCC-TNM staging system is the statistical bias because it is primarily based on data from single-center studies. Furthermore, the 7th and 8th AJCC-TNM staging systems have not been broadly validated in a large population-based sample, and validation studies have mostly been conducted in Europe [8, 9] and the United States [10], even though penile cancer is more common in southern Africa [1, 9, 10] and parts of Asia [1, 2].

The presence of lymphovascular embolization, perineural invasion, and the degree of differentiation are all considered prognostic indicators of survival for penile cancer patients [5, 7, 11–13]. However, the 8th AJCC-TNM staging system only incorporates these features into the T1 category, and some studies also showed that pathological indicators could be used to predict or influence predictions of the T2–3 stages [1, 2, 9]. In this study, we analyzed the differences between the T2 and T3 categories in the 8th AJCC-TNM staging system and discussed the predictive value of a modified staging system for T2–3 penile cancer.

## Patients and methods

### Patient selection

After this study received appropriate institutional review board approval (B2015-076-15 in China; CEP INCA 38/05 and 067/07 in Brazil), a training cohort comprising patients with penile cancer treated between January 2000 and March 2015 at 2 centers was developed. Lymphovascular embolization is defined as either tumor embolization within the endothelium-lined spaces that are bound by a thin wall or the absence of parietal smooth muscle fibers and red blood cells [14, 15]. The inclusion criteria were as follows: histologically confirmed penile squamous cell carcinoma, initial treatment of the primary tumor at one of the study centers, detailed patient information regarding demographic and tumor characteristics, and a subsequent follow-up of more than 1 month.

An external validation cohort of patients treated between January 2000 and March 2015 at 15 centers over four continents was also assembled. Eligible patients were selected based on the abovementioned standards.

The treatment protocols were discussed with each patient based on established guidelines [9, 10, 16–19]. The histopathological data were reviewed by an independent pathological committee, and all histopathological reports were based on the 8th AJCC-TNM staging system [2].

### Follow-up

The follow-up period for each patient began at the time of initial cancer diagnosis and ended either at the patient’s death or until March 2016. All patients underwent follow-up examinations every 3 months for the first 2 years after surgery, every 6 months in the 3rd and 4th years after surgery, and every year thereafter. Follow-up examination included imaging and physical examination of the penis and groin. Tissues from the glans were collected right after laser ablation or topical chemotherapy for histopathological examination to confirm the disease-free status. After potentially curative treatment for inguinal nodal metastases, computed tomography or magnetic resonance imaging was used to detect systemic disease. The cancer-specific survival (CSS) was calculated as the period from the date of initial cancer diagnosis until either the date of death due to cancer or the last known follow-up [20].

### Statistical analysis and proposed modified clinicopathological staging system

The statistical analyses were performed with SPSS version 20.0 (SPSS Institute, Chicago, IL, USA) and R 2.14.1 (http://www.r-project.org) with the survival and rms packages. Categorical variables were compared using the Chi square test. Kaplan–Meier plots were used to estimate CSS of the training and external validation cohorts, and survival curves were compared using the log-rank test. Because adjuvant therapies were not routinely administered to enrolled patients (which is usually recommended for patients with N2–3 disease), the role of these therapies was not evaluated in the multivariate analysis. The 8th N staging system includes lymph node metastasis laterality, the number of metastatic lymph nodes, extranodal extension, and pelvic lymph node metastasis. We also added lymphovascular embolization to our modified staging system. Therefore, these predictors were excluded from the multivariate analysis. We subdivided the T2 and T3 categories of the 8th AJCC-TNM staging system into the following two subcategories in the training cohort: T2 or T3 tumors without lymphovascular embolization (T2a and T3a) and tumors with lymphovascular embolization (T2b and T3b). A modified staging system was proposed. In this system, we subdivided the T2 and T3 categories of the 8th AJCC-TNM staging system into two new categories as follows: t2 tumors invade the corpus spongiosum and/or corpora cavernosa and/or urethra without lymphovascular invasion, and t3 tumors invade the corpus spongiosum and/or corpora cavernosa and/or urethra with lymphovascular invasion.

The accuracy of the staging systems was investigated using area under the receiver-operating characteristic curve (AUC) and Harrell’s concordance index (C-index). Bootstrap-corrected C-indexes (1000 samples of the same number from the original database) were used for internal validation, and the external validation cohort was used to validate the developed models. A two-sided *P* < 0.05 indicated statistical significance.

## Results

### Clinicopathological features

A training cohort with 411 treated patients and an external validation cohort with 436 patients were created. The median age at diagnosis was 53 years (range, 24–94 years) in the training cohort and 56 years (range, 18–93 years) in the external validation cohort. The detailed clinicopathological characteristics of the training and external validation cohorts are listed in Table 1.

**Table 1:** Clinical and pathological characteristics of the patients with penile cancer

Variable	Training cohort [cases (%)]	External validation cohort [cases (%)]
Total	411	436
Age, year, median (range)	53.0 (24.0–94.0)	56.0 (18.0–93.0)
Asia		
Mainland China	236 (57.4)	173 (39.7)
Taiwan, China	-	27 (6.2)
South America		
Brazil	175 (42.6)	-
Paraguay	-	166 (38.1)
Europe		
Germany	-	23 (5.3)
North America		
USA	-	47 (10.8)
T stage		
≤ T1^a^	158 (38.4)	143 (32.8)
T2	115 (28.0)	142 (32.6)
T3	119 (29.0)	142 (32.6)
T4	19 (4.6)	9 (2.1)
N stage		
N0	252 (61.3)	353 (81.0)
N1	53 (12.9)	30 (6.9)
N2	36 (8.8)	21 (4.8)
N3	70 (17.0)	32 (0.7)
M stage		
M0	403 (98.1)	430 (98.6)
M1	8 (1.9)	6 (1.4)
Grade		
G1-2	379 (92.2)	328 (75.2)
G3	32 (7.8)	108 (24.8)
Lymphovascular embolization		
Yes	70 (17.0)	93 (21.3)
No	341 (83.0)	343 (78.7)
Perineural invasion		
Yes	56 (13.6)	65 (14.9)
No	355 (86.4)	371 (85.1)
Modified staging system^b^		
≤ t1	158 (38.4)	143 (32.8)
t2	178 (43.3)	218 (50.0)
t3	56 (13.6)	66 (15.1)
t4	19 (4.6)	9 (2.1)

^a^≤ T1 including T0, Tis, Ta, and T1

^b^≤ t1 including t0, tis, ta, and t1

In the training cohort, 44 (10.7%) patients chose penis preservation and underwent either local excision or circumcision, 292 (71.0%) underwent partial penile amputation, and 71 (17.2%) underwent total penile amputation; however, 2 (0.5%) underwent laser therapy and 2 (0.5%) did not report primary tumor treatment (due to unknown reasons). In the external validation cohort, 14 (3.2%) patients chose penis preservation and underwent local excision or circumcision, 318 (72.9%) underwent partial penile amputation, and 80 (18.3%) underwent total penile amputation; however, 1 (0.2%) underwent laser therapy, and 23 (5.6%) did not report primary tumor treatment (due to unknown reasons).

A total of 15 patients with follow-up shorter than 1 month were excluded from the analysis. During follow-up, 92 patients in the training cohort died of penile cancer after a median of 18.0 (1.0–207.0) months, and 77 patients in the external validation cohort died of penile cancer after a median of 35.4 (1.0–349.7) months.

### Prognostic value of T stage

**Fig. 1:**
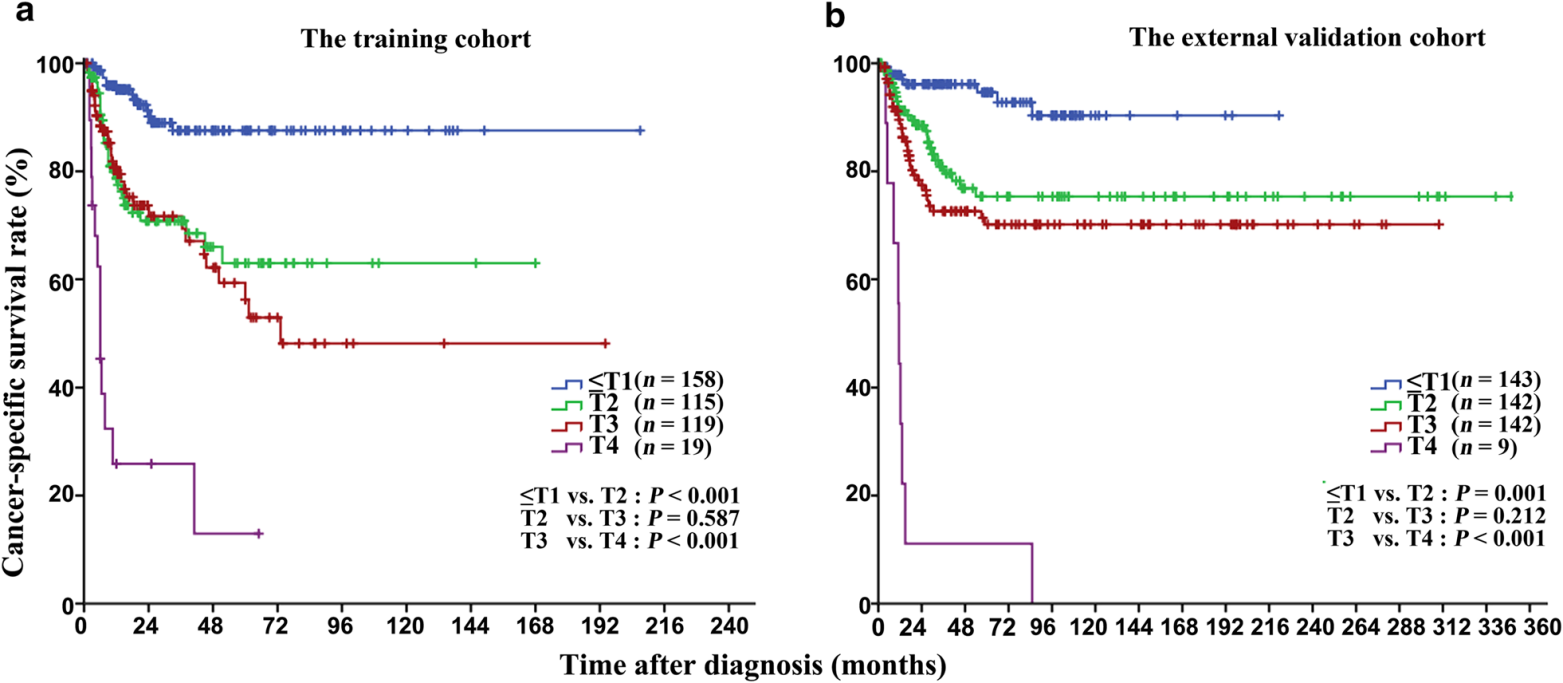
Kaplan-Meier cancer-specific survival (CSS) curves of patients with penile cancer at different T stages classified according to the 8th American Joint Committee on Cancer tumor–node–metastasis (AJCC-TNM) staging system. **a** In the training cohort, the 5-year CSS curves show no significant difference between patients with T2 and T3 diseases. **b** In the external validation cohort, the 5-year CSS curves also show no significant difference between patients with T2 and T3 diseases

The 5-year CSS rates are shown in Table 2. Based on the Kaplan–Meier analysis, no significant difference was observed in survival between patients with T2 and T3 diseases in both the training cohort (63.0% vs. 56.2%, *P* = 0.587, Fig. 1a) and the external validation cohort (75.3% vs. 70.1%, *P *= 0.212, Fig. 1b).

**Table 2:** The 5-year cancer-specific survival (CSS) rate of the patients with penile cancer

Variable	5-year CSS rate (%, 95% CI)			
	Training cohort	*P* value	External validation cohort	*P* value
T stage		< 0.001		< 0.001
≤ T1^a^	87.5 (81.0–94.0)		94.6 (90.0–99.1)	
T2	63.0 (51.0–75.0)		75.3 (66.5–84.1)	
T3	56.2 (42.7–69.7)		70.1 (61.7–78.5)	
T4	13.0 (0–33.8)		11.1 (0–31.7)	
N stage		< 0.001		< 0.001
N0	94.3 (90.8–97.8)		90.8 (87.3–94.3)	
N1	68.0 (50.6–85.4)		55.7 (29.2–82.2)	
N2	9.5 (0–21.8)		12.9 (0–29.4)	
N3	0		0	
M stage		< 0.001		< 0.001
M0	69.9 (63.8–76.0)		79.6 (75.1–84.1)	
M1	0		0	
Pathological grade		< 0.001		0.002
G1–2	71.6 (65.5–83.8)		81.4 (76.3–86.5)	
G3	18.7 (0–47.7)		69.8 (60.2–79.4)	
Lymphovascular embolization		< 0.001		< 0.001
Yes	30.8 (15.9–45.7)		50.0 (38.8–61.2)	
No	77.0 (70.9–77.0)		86.5 (82.2–90.8)	
Perineural invasion		< 0.001		0.010
Yes	40.3 (23.6–57.0)		65.7 (52.0–79.4)	
No	73.3 (67.0–79.6)		80.5 (75.8–85.2)	
Modified staging system		< 0.001		< 0.001
≤ t1^b^	87.5 (81.0–94.0)		94.6 (90.0–99.1)	
t2	69.2 (59.2–89.2)		82.5 (76.4–88.6)	
t3	33.4 (16.5–50.3)		42.9 (29.2–56.6)	
t4	13.0 (0–33.8)		11.1 (0–31.7)	

*CI* confidence interval

^a^≤ T1 includes T0, Tis, Ta, and T1

^b^≤ t1 includes t0, tis, ta, and t1

### Prognostic values of pathological indicators

**Fig. 2:**
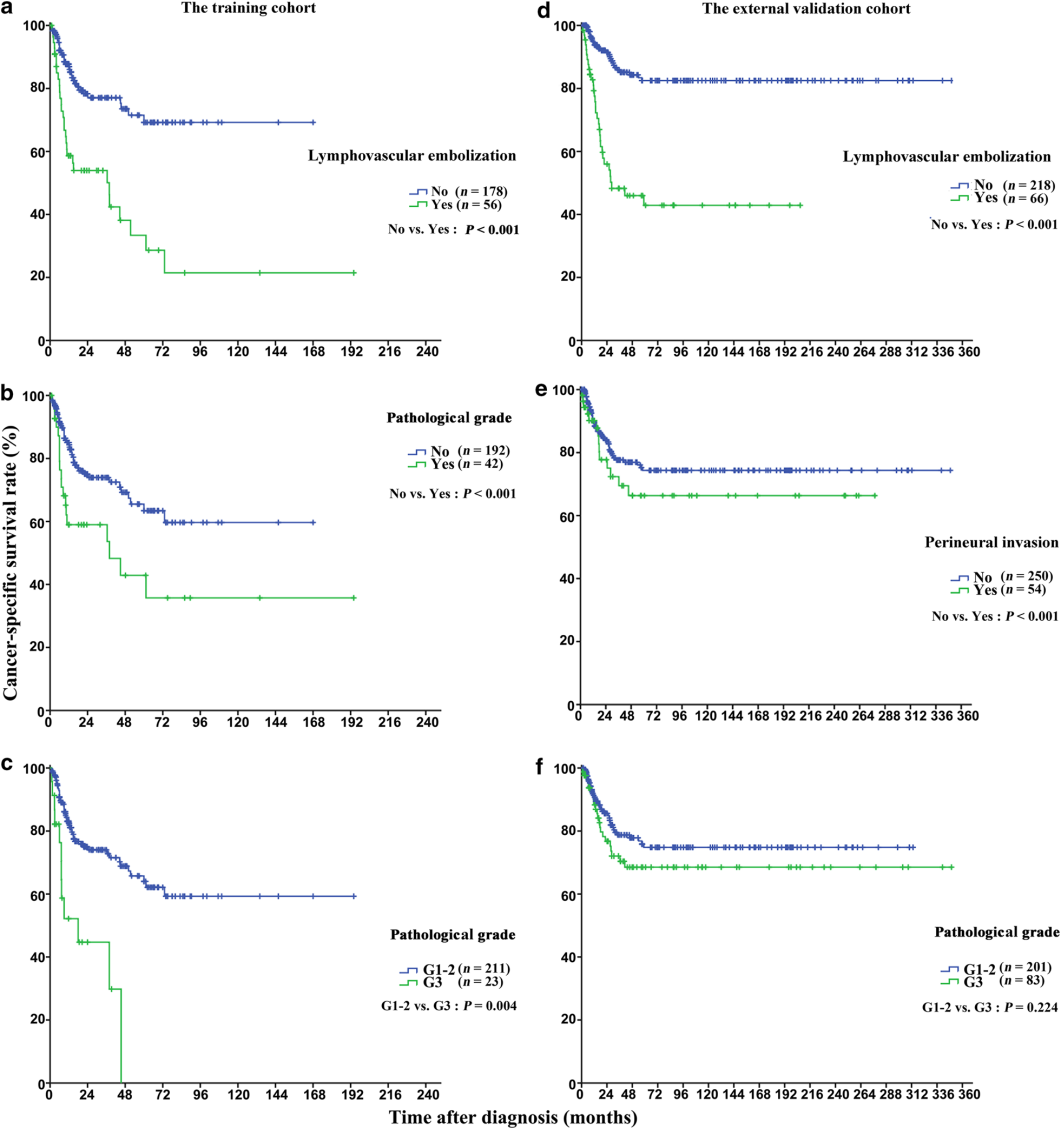
Kaplan-Meier CSS curves of patients with pT2–3 penile cancer. In the training cohort, shorter CSS was associated with lymphovascular embolization (**a**) (*P* < 0.001), perineural invasion (**b**) (*P* < 0.001), and pathological grade (**c**) (*P* = 0.004). In the external validation cohort, shorter CSS was associated with lymphovascular embolization (**d**) (*P* < 0.001) and perineural invasion (**e**) (*P* < 0.001), but not with pathological grade (**f**) (*P* = 0.224)

Survival was significantly related to lymphovascular embolization, perineural invasion, TNM stages, and pathological grade in both cohorts (Table 2). Furthermore, the corresponding survival rates of patients with T2–3 disease were significantly related to with lymphovascular embolization, perineural invasion, and pathological grade in the training cohort, whereas only lymphovascular embolization was associated with lymph node metastasis in the external validation cohort (Table 3, Fig. 2). After adjusting the variables, the multivariate analysis indicated that only lymphovascular embolization and N stage had a significant influence on CSS in both cohorts (Table 4).

**Table 3:** Relationships between pathological factors and lymph node metastasis of patients with penile cancer

Variable	Training cohort (*n* = 411)				External validation cohort (*n* = 436)			
	N0 [cases (%)]	N + [cases (%)]	χ^2^	*P*	N0 [cases (%)]	N + [cases (%)]	χ^2^	*P*
Lymphovascular embolization			12.12	< 0.001			61.32	< 0.001
Yes	30 (42.9)	40 (57.1)			49 (52.7)	44 (47.3)		
No	222 (65.1)	119 (35.0)			304 (88.7)	39 (11.3)		
Perineural invasion			11.20	< 0.001			1.54	0.214
Yes	23 (41.1)	33 (58.9)			49 (72.1)	16 (27.9)		
No	229 (64.5)	126 (35.5)			304 (81.9)	67 (18.1)		
Pathological grade			16.12	< 0.001			2.89	0.069
G1–2	243 (64.1)	136 (35.9)			272 (82.9)	56 (17.1)		
G3	9 (28.1)	23 (71.9)			81 (75.0)	27 (25.0)

**Table 4:** Multivariable Cox regression analyses for cancer-specific survival of the patients with penile cancer

Variable	Training cohort			External validation cohort		
	HR	95% CI	*P* value	HR	95% CI	*P* value
T stage (≤ T2 vs. ≥ T3)	1.513	0.975–2.347	0.065	1.830	1.123–2.980	0.015
N stage (N0 vs. N +)	11.502	6.155–21.494	< 0.001	13.541	7.774–23.588	< 0.001
M stage (M0 vs. M1)	1.448	1.1161–1.805	0.001	1.218	0.971–1.528	0.088
Pathological grade (G1–2 vs. G3)	1.250	0.671–2.329	0.481	0.894	0.533–1.500	0.894
Lymphovascular embolization (yes vs. no)	1.587	1.253–2.011	0.001	1.359	1.029–1.796	0.031
Perineural invasion (yes vs. no)	1.088	0.841–1.407	0.522	1.242	0.944–1.634	0.121

*HR* hazard ratio, *CI* confidence interval

**Fig. 3:**
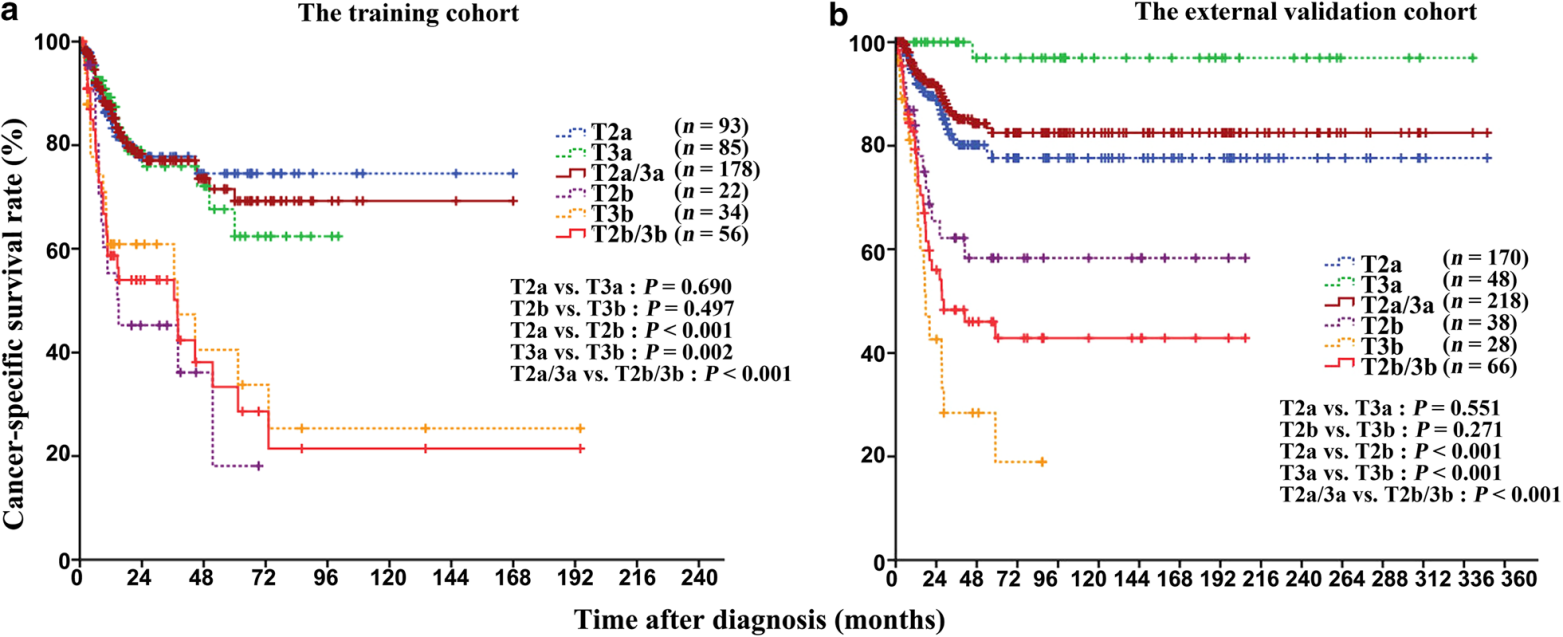
Kaplan-Meier CSS curves of patients with T2–3 penile cancer classified according to the 8th AJCC-TNM staging system. **a** In the training cohort, the CSS was significantly longer in T2 patients without lymphovascular embolization (T2a) than in those with lymphovascular embolization (T2b) (*P* < 0.001) and significantly longer in T3 patients without lymphovascular embolization (T3a) than in those with lymphovascular embolization (T3b) (*P* = 0.002). **b** Similar results were observed in the external validation cohort

In the training cohort, the 5-year CSS rate was significantly higher in T2a patients than in T2b patients (74.5% vs. 18.1%, *P* < 0.001) and significantly higher in T3a patients than in T3b patients (65.4% vs. 25.3%, *P* = 0.002). However, the 5-year CSS rates of T2a and T3a patients (*P* = 0.690) as well as those of T2b and T3b patients were similar (*P* = 0.497). When stratifying the training cohort by lymphovascular embolization, T2a/3a patients had longer CSS than did T2b/3b patients (*P* < 0.001, Fig. 3a). Similar results were observed in the external validation cohort (Fig. 3b).

### The modified staging system

**Fig. 4:**
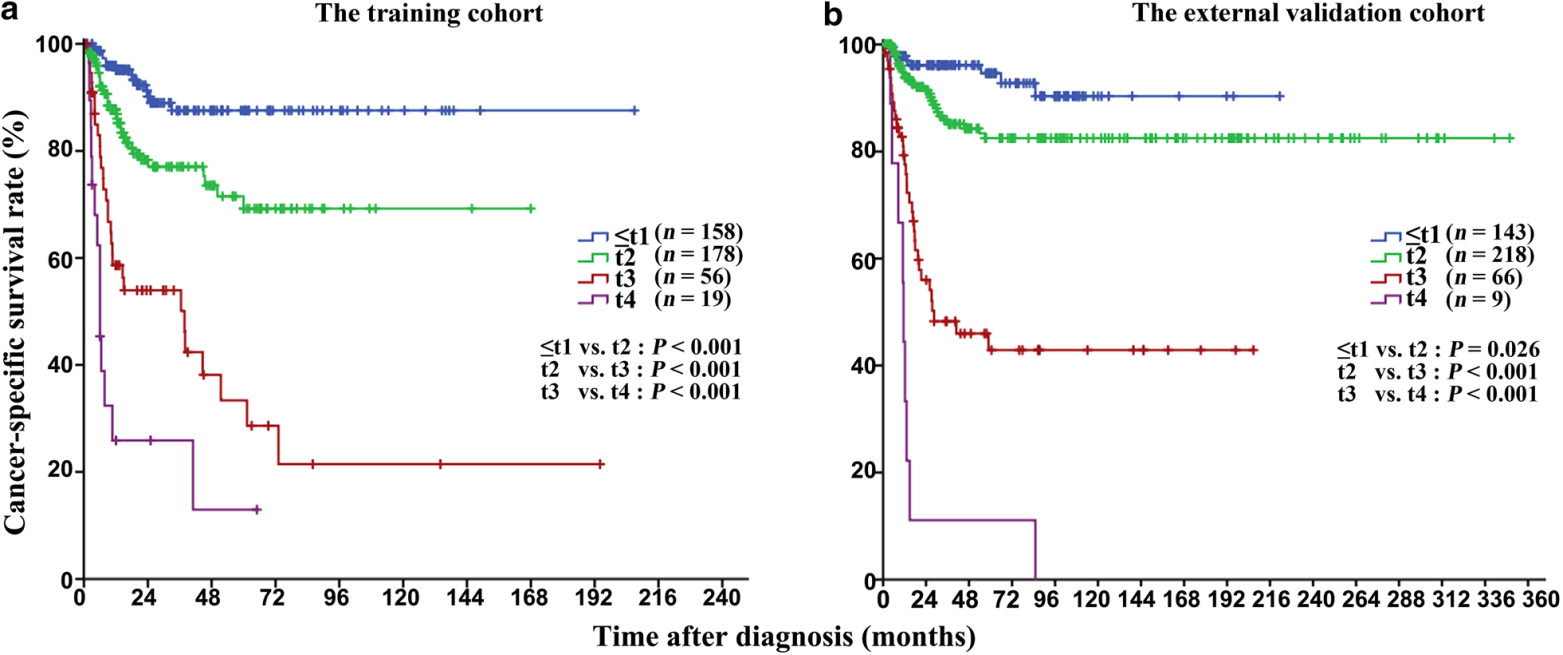
Kaplan-Meier CSS curves of patients with penile cancer at different t stages classified according to the modified staging system. The CSS was significantly different among all categories in both the training cohort (**a**) and the external validation cohort (**b**)

**Fig. 5:**
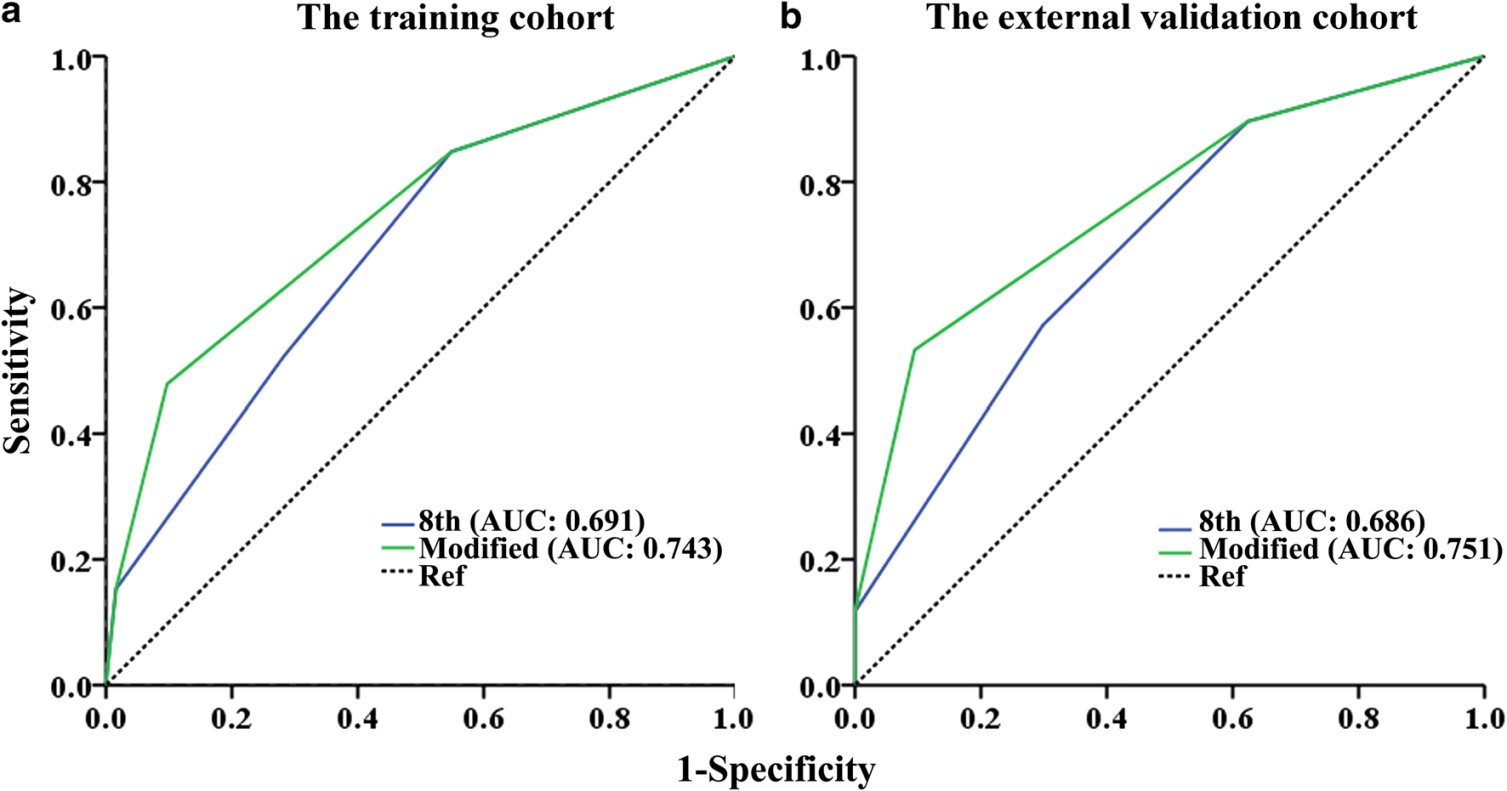
Receiver operating characteristic (ROC) curve analysis of different T stages classified according to the 8th AJCC-TNM staging system and the modified staging system for the prediction of CSS. **a** Training cohort, **b** external validation cohort. The dashed line from the left bottom to the top right corners represents a random guess regardless of the positive and negative base rates. *AUC* area under the ROC curve, *8th* the 8th AJCC-TNM staging system, *modified* the modified staging system, *Ref* reference line

Using the modified staging system, the 5-year CSS rates were 87.5% (95% confidence interval [CI] 81.0%–94.0%), 69.2% (95% CI 59.2%–89.2%), 33.4% (95% CI 16.5%–50.3%), and 13.0% (95% CI 0–43.8%) for t1 to t4 patients, respectively. This modified staging system provided an improved prognostic stratification with significant differences in CSS among all the categories (all *P* < 0.001; Fig. 4a). Similar results were observed in the external validation cohort (Fig. 4b). The AUC of the modified staging system was significantly larger than that of the 8th AJCC-T staging system in both cohorts (Fig. 5 and Table 5). Moreover, the modified staging system showed a higher C-index and a higher bootstrap value than did the 8th AJCC-T staging system (Table 5).

**Table 5:** The accuracy of the staging systems in predicting prognosis of patients with penile cancer

Staging system	Training cohort			External validation cohort	
	AUC	C-index	Bootstrap C-index	AUC	C-index
8th AJCC-T staging system	0.691	0.696	0.697	0.698	0.686
Modified staging system	0.743	0.739	0.738	0.765	0.751

*AUC* area under the receiver operating characteristic curve

## Discussion

The pT2–3 penile cancer is heterogeneous, and a modified staging system that includes lymphovascular embolization may help improve the prognostic accuracy for these men. We evaluated the prognostic value of the 8th AJCC-TNM staging system in a large group of patients with penile cancer from four continents. Although we confirmed the importance of the T status as a prognostic factor, our study also showed the potential for improvement of the AJCC-TNM staging system.

Penile cancer is a relatively uncommon urological malignancy [1, 2, 9, 10, 17]. Primary penile cancer has an overall incidence of < 1.00/100,000 males in developed countries [1, 2]. In contrast, the incidence is much higher in undeveloped areas, such as regions in Africa [1, 9, 10] and Asia [1, 2]. Therefore, references with more than 500 cases are rarely cited by the European Association of Urology (EAU) and National Comprehensive Cancer Network (NCCN) for TNM staging [1, 2, 9, 19]. Further evaluations with larger sample sizes are needed to validate the current TNM staging system. The results of our study of 847 men are consistent with the results of previous studies that have demonstrated the overall usefulness of the TNM staging system [4, 21]. However, we found room for improvement in survival estimates among subgroups of T2–3 patients.

The most important prognostic indicators of penile cancer are pathological factors [1, 2, 9, 19]. Lymphovascular embolization is defined as either tumor embolization within the endothelium-lined spaces that are bound by a thin wall or the absence of parietal smooth muscle fibers and red blood cells [14, 15]. The presence of lymphovascular embolization is an important prognostic indicator of survival in patients with penile cancer [7, 11, 13]. However, the EAU guidelines only incorporate lymphovascular invasion and classify penile cancer with lymphovascular invasion into the T1 category [10]. In our database, lymphovascular invasion was markedly associated with poor survival.

Distinguishing between the prognoses for T2 patients and T3 patients is difficult [6, 11, 22]. Leijte et al. [6] evaluated a large cohort of 489 patients in the Netherlands and demonstrated that patients with corpus spongiosum/cavernosum and urethra/prostate involvement exhibited similar survival (*P* = 0.570). In the study by Graafland et al. [11], the 5-year CSS rates of patients with pT2 and pT3–4 diseases were 60.0% and 59.0%, respectively. Ravi [22] noted 3-year CSS rates of 69.9% and 100.0% in patients with T2 and T3 diseases. Furthermore, the estimates of occult metastasis in T2 and T3 patients were similar [6, 11, 15, 23]. The 8th AJCC-TNM staging system for penile cancer redefined the T2 and T3 categories based on the presence or absence of corpus spongiosum and cavernosa invasion [1, 2]. However, we found that the prognoses of patients with T2 and T3 disease classified according to the 8th AJCC-TNM staging system were similar. Therefore, we stratified pT2–3 tumors by the presence of lymphovascular embolization and observed different characteristics between these subgroups in the training and external validation cohorts.

To improve the prognostic prediction, we proposed a modified staging system, which showed improved accuracy in survival prediction and was validated using multicenter data obtained globally. Differences existed between the training and external validation cohorts regarding the number of T3 patients, grade status, and follow-up period. We presume that the differences may be related to races or regions and that data heterogeneity may lead to selection bias [1, 2]. However, this heterogeneity may help confirm that the modified staging system has universal applicability across a heterogeneous population of patients from different regions. Accurate staging with appropriate subgroup classification of the disease is the first step in optimizing treatment and predicting outcomes [24].

The present study had some limitations. First, the data collection was retrospective and covered a long study period, which required lengthy follow-ups. The histopathological data were reviewed by an independent pathological committee. We acknowledge that the slides could not be reviewed again by a single urological pathologist, which limited the value of the study. Second, some important information was not reported in this study. Our study population was selected in a 16-year period from multiple centers. The lymph node dissection details of both cohorts were not analyzed because of the varying treatment standards during the study period. However, we ensured that the therapeutic principles were in accordance with the EAU and NCCN guidelines for penile cancer. Clinical stage and some pathological factors (e.g., tumor growth and depth, histological subtype, positive surgical margin, and front invasion) of the primary tumor that could influence the survival rates were not included in the study. Our study included a total of 847 patients who were treated at 17 centers from four continents; thus, some pathological factors were collected in a fragmentary manner. To ensure that statistical bias was minimized and to maximum the sample size of lymphovascular invasion cases, we did not perform a detailed analysis of these predictors. Third, our study contained treatment diversity. In particular, adjuvant therapies and the treatment courses varied, and adjuvant therapies and pelvic lymphadenectomy might affect other parameters. Pelvic lymphadenectomy was not routinely performed prior to 2009 because it was not recommended by the guidelines as the standard treatment for penile cancer [16–18]. Some patients who should have been treated with adjuvant therapies chose not to receive these regimens for various reasons [3, 4]. Because of this variability, we did not report the prognostic value of adjuvant therapies in this study. However, we believe that this type of analysis will be important in future validation studies with larger data sets. This study can be considered exploratory rather than hypothesis-driven, and thus comparisons between the various models were not explored further. We also deem it necessary to intensively study this modified staging system in the future.

## Conclusions

A modified clinicopathological staging system that stratifies patients with pT2–3 penile squamous cell cancer by lymphovascular embolization may increase the accuracy of survival prediction.

## Abbreviations

AJCC-TNM: American Joint Committee on Cancer tumor–node–metastasis
AUC: area under curve
C-index: Harrell’s concordance index
CSS: cancer-specific survival
EUA: European Urology Association
NCCN: National Comprehensive Cancer Network

## References

[1] Hakenberg OW, Comperat EM, Minhas S, Necchi A, Protzel C, Watkin N. EAU guidelines on penile cancer. http://www.uroweborg/guideline/penile-cancer/, Accessed Jan 1 2017. 10.1016/j.eururo.2014.10.017 25457021

[2] Amin. American joint committee on cancer. AJCC cancer staging manual. 8th ed. https://cancerstaging.Org/pages/default.Aspx. Accessed Jan 1 2017.

[3] Zhu Y, Ye DW, Yao XD, Zhang SL, Dai B, Zhang HL (2011). New n staging system of penile cancer provides a better reflection of prognosis. J Urol.

[4] Li ZS, Yao K, Chen P, Wang B, Chen JP, Mi QW (2015). Modification of n staging systems for penile cancer: a more precise prediction of prognosis. Br J Cancer.

[5] Thuret R, Sun M, Abdollah F, Budaus L, Lughezzani G, Liberman D (2011). Tumor grade improves the prognostic ability of american joint committee on cancer stage in patients with penile carcinoma. J Urol.

[6] Leijte JA, Gallee M, Antonini N, Horenblas S (2008). Evaluation of current TNM classification of penile carcinoma. J Urol.

[7] Geng JH, Huang SP, Huang CY (2015). Prognostic factors in taiwanese patients with penile-invasive squamous cell carcinoma. Kaohsiung J Med Sci.

[8] Torbrand C, Wigertz A, Drevin L, Folkvaljon Y, Lambe M, Hakansson U (2017). Socioeconomic factors and penile cancer risk and mortality; a population-based study. BJU Int.

[9] Hakenberg OW, Comperat EM, Minhas S, Necchi A, Protzel C, Watkin N (2015). EAU guidelines on penile cancer: 2014 update. Eur Urol.

[10] Pizzocaro G, Algaba F, Horenblas S, Solsona E, Tana S, Van Der Poel H (2010). EAU penile cancer guidelines 2009. Eur Urol.

[11] Graafland NM, van Boven HH, van Werkhoven E, Moonen LM, Horenblas S (2010). Prognostic significance of extranodal extension in patients with pathological node positive penile carcinoma. J Urol.

[12] Emerson RE, Ulbright TM, Eble JN, Geary WA, Eckert GJ, Cheng L (2001). Predicting cancer progression in patients with penile squamous cell carcinoma: the importance of depth of invasion and vascular invasion. Mod Pathol.

[13] Guimaraes GC, Cunha IW, Soares FA, Lopes A, Torres J, Chaux A (2009). Penile squamous cell carcinoma clinicopathological features, nodal metastasis and outcome in 333 cases. J Urol.

[14] Ornellas AA, Nobrega BL, Wei Kin Chin E, Wisnescky A, da Silva PC, de Santos Schwindt AB (2008). Prognostic factors in invasive squamous cell carcinoma of the penis: analysis of 196 patients treated at the Brazilian national cancer institute. J Urol.

[15] Zhu Y, Zhang HL, Yao XD, Zhang SL, Dai B, Shen YJ (2010). Development and evaluation of a nomogram to predict inguinal lymph node metastasis in patients with penile cancer and clinically negative lymph nodes. J Urol.

[16] Algaba F, Horenblas S, Pizzocaro-Luigi Piva G, Solsona E, Windahl T, European Association of Urology (2002). EAU guidelines on penile cancer. Eur Urol.

[17] Solsona E, Algaba F, Horenblas S, Pizzocaro G, Windahl T, European Association of Urology (2004). EAU guidelines on penile cancer. Eur Urol.

[18] Hegarty PK (2007). EAU guidelines for management of penile cancer. Indian J Urol.

[19] Flaigi TW, Spiess PE, Bangs R, Boorjian SA, Buyyounouski MK, Efstathiou, et al. NCCN clinical practice guidelines in oncology (nccn guidelines) penile cancer. https://www.Nccn.Org/professionals/physician_gls/f_guidelines.Asp#penile. Accessed Jan 1 2016.

[20] Cabibbo G, Maida M, Genco C, Parisi P, Peralta M, Antonucci M (2012). Natural history of untreatable hepatocellular carcinoma: a retrospective cohort study. World J Hepatol.

[21] Li ZS, Yao K, Chen P, Zou ZJ, Qin ZK, Liu ZW (2014). Disease-specific survival after radical lymphadenectomy for penile cancer: prediction by lymph node count and density. Urol Oncol.

[22] Ravi R (1993). Correlation between the extent of nodal involvement and survival following groin dissection for carcinoma of the penis. BJU Int.

[23] Al-Najar A, Alkatout I, Al-Sanabani S, Korda JB, Hegele A, Bolenz C (2011). External validation of the proposed t and n categories of squamous cell carcinoma of the penis. Int J Urol.

[24] Kim MK, Warner RR, Roayaie S, Harpaz N, Ward SC, Itzkowitz S (2013). Revised staging classification improves outcome prediction for small intestinal neuroendocrine tumors. J Clin Oncol.

